# Does Exercise Improve Cognitive Performance? A Conservative Message from Lord's Paradox

**DOI:** 10.3389/fpsyg.2016.01092

**Published:** 2016-07-21

**Authors:** Sicong Liu, Jean-Charles Lebeau, Gershon Tenenbaum

**Affiliations:** Department of Educational Psychology and Learning System, Florida State UniversityTallahassee, FL, USA

**Keywords:** exercise intervention, cognition, gain score analysis, ANCOVA, experimental group equivalence, false positive error, review

## Abstract

Although extant meta-analyses support the notion that exercise results in cognitive performance enhancement, methodology shortcomings are noted among primary evidence. The present study examined relevant randomized controlled trials (RCTs) published in the past 20 years (1996–2015) for methodological concerns arise from Lord's paradox. Our analysis revealed that RCTs supporting the positive effect of exercise on cognition are likely to include Type I Error(s). This result can be attributed to the use of gain score analysis on pretest-posttest data as well as the presence of control group superiority over the exercise group on baseline cognitive measures. To improve accuracy of causal inferences in this area, analysis of covariance on pretest-posttest data is recommended under the assumption of group equivalence. Important experimental procedures are discussed to maintain group equivalence.

## Introduction

Does exercise enhance cognitive functioning in human beings? Meta-analyses have provided support for the beneficial effect of exercise on cognitive performance with effect sizes (*g*) ranging from 0.097 for acute exercise (Chang et al., 2012) to 0.158 for chronic exercise (Smith et al., 2010). Additionally, some authors have reported on several underlying mechanisms by considering evidence from behavioral and psychophysiological studies (for a review, see Hillman et al., 2008). These arguments seem to offer convincing evidence that exercise results in cognitive performance enhancement. The present study takes a critical perspective on this conclusion by assessing methodological characteristics of relevant evidence.

The most relevant evidence comes from exercise-cognition randomized controlled trials (RCT). First, these RCTs are considered clinical trials. According to World Health Organization (2015, para. 3) and the International Committee of Medical Journal Editors (Laine et al., 2007, p. 275), a clinical trial “is any research study that prospectively assigns human participants or groups of humans to one or more health-related interventions to evaluate the effects on health outcomes.” Second, RCT is generally regarded as the best design for testing causal relationship because it makes group equivalence likely on all covariates (Freedman et al., 2007; Torgerson, 2009).

Several Exercise-cognition RCTs' findings support the causal relationship between exercise and cognition. For example, Chang et al. (2012) reported a larger effect size from RCTs (*d* = 0.19) compared to those from either quasi-experimental or observational designs (*d* = −0.02 and *d* = −0.14, respectively). These results have led some authors to conclude that exercise benefits cognition in a population ranging from children to older adults. Although such message is exciting, as Rubin (1974) cautioned, the relevance of evidence to answering research questions is not solely determined by the choice of research design but many other factors. Guided by this message, we examined exercise-cognition RCTs published in the past 20 years for potential methodological shortcomings.

### Why are errors possible

When analyzing pretest-posttest data from RCTs, researchers typically apply two group-comparison strategies to draw causal inferences: analysis of covariance and gain score analysis (Vickers and Altman, 2001; Van Breukelen, 2006). *Analysis of Covariance* (ANCOVA)^1^ refers to the approach where posttest scores are compared between groups, adjusting for baseline scores (as covariates in the linear model). Assuming baseline group equivalence, *Analysis of Partial Variance* is a parallel of this strategy (Cohen et al., 2013). The alternative approach, *Gain Score Analysis* (GSA), considers the gain score (i.e., posttest minus pretest) as the criterion for group comparison. Forms of GSA include repeated-measures analysis of variance (RM ANOVA), gain score *t*-test, and ANOVA of gain score, among others. Researchers' choice between ANCOVA and GSA often leads to disparate conclusions, an inconsistency historically termed “Lord's Paradox” (Lord, 1967).

Lord's paradox generated a lasting research effort and a consensus was reached among methodologists. The consensus is that, as long as baseline group equivalence is likely by randomization (such as in a RCT design), investigators should choose ANCOVA in drawing causal conclusions, because ANCOVA has a higher testing power and unbiased effect estimate compared to GSA (Cronbach and Furby, 1970; Huck and McLean, 1975; Holland and Rubin, 1983; Miller and Chapman, 2001; Senn, 2006; Van Breukelen, 2006). However, when baseline group equivalence is unlikely (such as in a quasi-experimental design), none of the statistical procedures enables to “control for” such a flaw, and thus no causal inferences should be attempted (Campbell and Stanley, 1963; Lord, 1967; Cronbach and Furby, 1970; Meehl, 1970; Senn, 2006; Van Breukelen, 2006). To reiterate previous points with an analogy, perfect dishes (“causal inferences”) come from fresh raw food (“baseline group equivalence”) and skillful cooking (“ANCOVA”), whereas no perfect dishes can be made from non-fresh food (“baseline group non-equivalence”) irrespective of how skillful the cook is.

Given Lord's paradox conclusion, strong evidence for causal inferences can be obtained only if (a) baseline group equivalence is likely, and (b) pretest-posttest data are analyzed using ANCOVA. In practice, researchers never know with certainty that a given RCT has baseline group equivalence, but they can ascertain baseline group non-equivalence when group baseline measures show statistical differences. Assuming that baseline group equivalence is achieved by identifying no baseline group differences on any baseline measures (which is a likely portrait of a given RCT, at least on baseline measures statistically tested), researchers should choose ANCOVA over GSA when comparing groups.

One advantage of ANCOVA over GSA is an increased power. Originally, ANCOVA was not developed to “control” for anything but to enhance the testing power of independent variables (Miller and Chapman, 2001). For instance, assuming identical within-group variance between pretest and posttest, Van Breukelen (2006) quantified that ANCOVA requires only 75% of the sample size of ANOVA of gain score (i.e., one form of GSA) to detect the same effect when the pretest-posttest correlation is 0.50. The other advantage of ANCOVA over GSA has to do with effect estimate accuracy. Specifically, ANCOVA produces the unbiased effect estimate, whereas GSA can generate under- or over- estimated effect size depending on the situation of baseline group imbalance (Vickers and Altman, 2001).

Baseline group imbalance is the descriptive difference between groups on baseline measures. If an exercise-cognition RCT has only two groups (i.e., one control and one exercise group), the control group and the exercise group have an equal chance to perform better than the other descriptively on a cognitive task at baseline. The interpretation of “better” is task specific. For instance, a shorter reaction time (RT) is better in simple reaction time tasks (e.g., Stroop Color), whereas a larger value is better in time-limited memory tasks (e.g., Digit Symbol). If the control group has baseline superiority (*control-BS*) by having, for instance, a shorter RT than that of the exercise group on the Stroop Color task, the adoption of GSA will lead to an over-estimate of exercise's benefits on cognition. Conversely, baseline exercise group superiority (*exercise-BS*) will generate an under-estimated effect with the GSA method (Vickers and Altman, 2001).

Baseline measures are usually negatively correlated with gain scores (Cronbach and Furby, 1970; Knapp and Schafer, 2009), a phenomenon known as “regression to the mean” (Galton, 1886; Bland and Altman, 1994). In such instances, the bias due to GSA's failure to account for baseline group imbalance can be larger. As a consequence, the Type I error (i.e., false positive) from control-BS and Type II error (i.e., false negative) from exercise-BS are likely to happen when using GSA. For example, Bland and Altman (2011) reported that comparing a baseline with a follow-up separately in each group by using *t*-test (i.e., one form of GSA) could raise the actual alpha level to be as high as 0.50 when comparing two groups and 0.75 when comparing three groups, depending on the power of a specific test. To make things worse, Bland and Altman's results were based on one outcome measure. When an exercise-cognition RCT assesses the effect of exercise on multiple cognitive measures (which is often the case), the practice of having a presumable false positive threshold (e.g., α = 0.05) could turn meaningless.

### How to test for possible errors

Rather than assessing the effect of exercise on cognition by considering potential moderators, a procedure common to meta-analytic studies, the focus of the present study was to determine whether exercise-cognition RCTs published in the past 20 years (1996–2015) involve false positives or false negatives due to GSA application in pretest-posttest data analysis. We provided a simple test to achieve this goal. Because group assignment was random, one would expect an equal chance for control-BS and exercise-BS on a certain cognitive measure. In other words, across all RCTs in our review, we expect half RCTs to show control-BS and the other half to have exercise-BS. In terms of a probability distribution, if we assume that *X* represents the number of RCTs showing control-BS, we would expect the probability of observing *X*, P (*X*), to follow a binomial distribution:

$$\begin{array}{l} P\left(X\right)\sim \text{Binomial}\left(n,k\right) \end{array}$$

where *n* represents the total number of RCTs examined and *k* symbolizes the expected probability (*k* = 0.5) of getting control-BS in a given exercise-cognition RCT^2^. Similarly, if researchers select randomly between GSA and ANCOVA, we should expect the group comparison strategy to follow the same binomial distribution with the only difference being that *X* is representing the number of RCTs employing GSA.

In order to detect possible false positive and/or negative errors among exercise-cognition RCTs using GSA, we must check for independence between baseline group imbalance (i.e., control-BS vs. exercise-BS) an statistical significance test result (i.e., significant vs. non-significant). If baseline group imbalance were independent to statistical significance test result, we would expect *X*, representing the number of RCTs using GSA that showed control-BS, to continue following the binomial distribution when conditioned on statistical test result. Assuming that *Y* stands for the statistical test result that has two possible outcomes (i.e., significant or non-significant), we will have the following conditional binomial distribution:

$$\begin{array}{l} \text{P}\left(X|Y\right)\;\sim \text{ Binomial}\left(n|Y,\;k\right) \end{array}$$

where *n* is the total number of RCTs using GSA method and *k* still takes the value of 0.5.

To summarize, we had three hypotheses in the present study. First, we hypothesized that, among all the RCTs, half of them should demonstrate control-BS and the other half should show exercise-BS due to randomization. Second, we hypothesized that researchers, as a group, selected between GSA and ANCOVA without preference, and therefore half of the RCTs should employ GSA and the other half should use ANCOVA as a group-comparison strategy. Lastly, we hypothesized that, when GSA-RCTs are counted separately based on whether they are positive (i.e., include at least one significant finding) or negative (i.e., include no significant findings), more control-BS (than exercise-BS) GSA-RCTs should be found in positive GSA-RCTs, whereas more exercise-BS (than control-BS) GSA-RCTs should be found in negative GSA-RCTs.

## Methods

### Literature search and inclusion criteria

The second author (J.-C. L.) conducted a literature search in April and May 2015 using SPORTDiscus, Web of Science, and Google Scholar databases. The search strategy utilized the following key words within full documents: (*exercise* OR *physical activity*) AND (*cognition* OR *cognitive performance*) AND *randomized controlled trial*. A manual search of reference list from key studies (e.g., meta-analysis) was also performed. The first author (S. L.) screened studies by title and abstract, then by full documentation. Trial authors were contacted when required information was missing. In total, 38 RCTs were considered for coding. However, five articles were excluded because they were missing information and corresponding authors were unable to respond to our request by July 1, 2015. The final set of studies consisted of 33 exercise-cognition RCTs.

**Figure 1:**
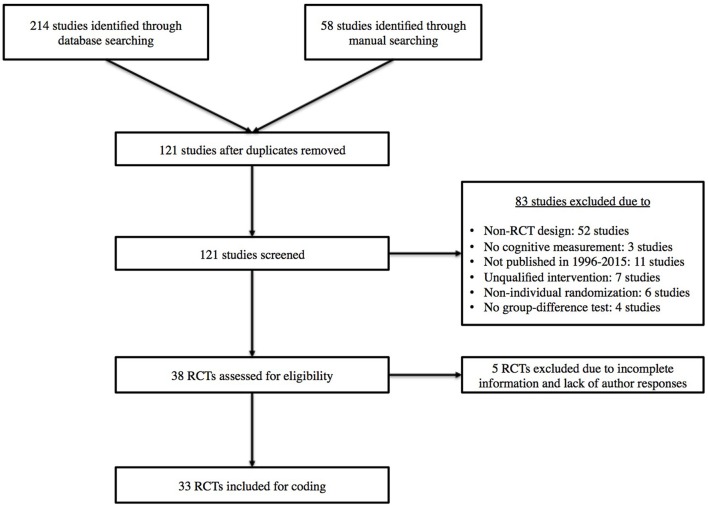
**Flowchart of study selection**.

The following inclusion criteria were applied to the exercise-cognition RCTs: (a) studies were published between January1996 and May 2015, (b) randomization is evident at the individual level, (c) the design included pre- and post-intervention measures on cognitive tasks such as perception, intelligence, academic achievement, memory, executive function, and cognitive impairment, (d) exercise intervention focused on aerobic, resistance training, or a combination of both, (e) studies included a passive control (e.g., waiting list), an active control (that can have a cognitive, physical, or social focus), or a combination of both (see Scherder et al., 2005), and (f) group differences were tested on cognitive measures. If multiple exercise intensities were used within an RCT, we regarded the group receiving the highest intensity as the exercise group and compared it to the control group. For example, if an RCT has two exercise groups (e.g., participants exercising at 60 and 70% of their VO_2max_) and a reading control group, the group exercising at 70% VO_2max_ was selected as the treatment group and was compared to the control group. In addition, if the two exercise groups differed in exercise modality (i.e., aerobic training and resistance training), we compared each of these exercise groups to the control group, respectively, and the results were coded under a given RCT. Furthermore, if multiple interventions were included and at least one of the groups received an intervention focusing on elements other than exercise (e.g., cognitive training), only the exercise group was considered as a treatment group and was compared to the control group. Finally, if multiple follow-up measurements were available after the intervention period, we chose the immediate post-intervention measurement as the post-test measure. Details of the literature search and study selection were shown in a flowchart (Figure 1).

### Coding and reliability

The first two authors discussed and settled coding variables to be included in the coding sheet. One author (S. L.) independently coded all the studies. The coded variables focused on the information relevant to the focus of the study, which is to check potential Type I and Type II errors in exercise-cognition RCTs. Therefore, for every cognitive task, we coded the targeted cognitive process (e.g., executive functioning), baseline group imbalance (control-BS vs. exercise-BS), and statistical test result (significant vs. non-significant). Other key methodological information were also coded including (a) group-comparison strategy in pretest-posttest data analysis (ANCOVA vs. GSA), (b) the form of control (passive vs. active), (c) the presence or absence of randomization procedure, (d) testing baseline group equivalence on cognitive measure(s), (e) the use of blinding procedures (i.e., single-, double-, or triple-blind), (f) explicit inclusion of intention-to-treat (ITT) analysis, (g) presence of *a priori* power analysis, (h) total participant number and number of groups (enabling participant number per group to be calculated), and (i) the presence or absence of pre-registering the trial. Table 1 displays the coded information for each study included.

**Table 1:** **Study coding sequenced by group comparison strategy and study positivity**.

**Authors and Year**	**Grp. (T/C)**	**Sig**.	**Anal**.	**Control**	**Random**	**Test Base**.	**Blind**	**ITT**	**Power**	***N* (Grp. #)**	**Prereg**.
Williamson et al., 2009	C/C	N	ANCOVA	A-Cog.	N	N	Single	N	Y	102(2)	Y
Scherder et al., 2005	E/E	Y	ANCOVA	Both	N	Y	Single	N	N	43(3)	N
Lautenschlager et al., 2008	E/E	Y	ANCOVA	A-Cog.	Y	Y	Single	Y	Y	170(2)	Y
Liu-Ambrose et al., 2010	C/C	Y	ANCOVA	A-Phy.	Y	N	Single	Y	Y	155(3)	Y
Davis et al., 2011	E/E	Y	ANCOVA	P	N	N	Single	Y	Y	171(2)	Y
Nagamatsu et al., 2012	E/E	Y	ANCOVA	A-Phy.	N	N	Single	N	N	86(3)	Y
Okumiya et al., 1996	E/E	N	GSA	P	N	Y	Single	N	N	42(2)	N
Lemmink and Visscher, 2005	E/E	N	GSA	A-Cog.	N	N	N	N	N	16(2)	N
Foley et al., 2008	E/E	N	GSA	A-Phy.	N	Y	N	Y	N	20(2)	N
Krogh et al., 2009	E/E	N	GSA	A-Phy.	Y	N	Single	Y	N	165(3)	Y
Kimura et al., 2010	E/E	N	GSA	A-Cog.	N	Y	Single	N	N	171(2)	N
Varela et al., 2012	C/C	N	GSA	A-Mix	N	N	Single	Y	N	68(3)	N
Ruscheweyh et al., 2011	C/C	N	GSA	P	N	N	Single	N	N	62(3)	N
Linde and Alfermann, 2014	E/E	N	GSA	P	Y	Y	Single	Y	N	70(4)	N
Ruiz et al., 2015	E/E	N	GSA	A-Mix	N	Y	Single	Y	N	40(2)	N
Williams and Lord, 1997	E/E	Y	GSA	P	N	Y	N	N	N	187(2)	N
Emery et al., 1998	C/C	Y	GSA	P	Y	N	N	N	N	79(2)	N
Erickson et al., 2011	E/E	Y	GSA	A-Phy.	N	N	Single	N	N	120(2)	N
Bakken et al., 2001	C/C	Y	GSA	P	N	N	N	N	N	15(2)	N
Kramer et al., 2001	C/C	Y	GSA	A-Phy.	N	N	N	N	N	124(2)	N
Fabre et al., 2002	C/C	Y	GSA	A-Soc.	N	Y	N	N	N	32(4)	N
Netz et al., 2007	C/C	Y	GSA	A-Cog.	N	Y	Single	N	N	59(3)	N
Busse et al., 2008	C/C	Y	GSA	P	N	N	N	N	N	31(2)	N
Chang and Etnier, 2009	C/C	Y	GSA	A-Cog.	N	N	N	N	N	41(2)	N
Barella et al., 2010	E/C	Y	GSA	A-Soc.	N	N	N	N	N	40(2)	N
Muscari et al., 2010	C/C	Y	GSA	A-Cog.	N	Y	Single	Y	Y	120(2)	N
Ellemberg and St-Louis-Deschênes, 2010	N/N	Y	GSA	A-Cog.	N	N	N	N	N	72(2)	N
Kamijo et al., 2011	C/C	Y	GSA	P	N	N	N	N	N	43(2)	N
Chang et al., 2011	C/C	Y	GSA	A-Cog.	N	Y	N	N	Y	42(2)	N
Hopkins et al., 2012	C/C	Y	GSA	P	N	N	N	N	N	75(4)	N
Maki et al., 2012	E/E	Y	GSA	A-Cog.	N	Y	N	Y	N	150(2)	N
Liu-Ambrose et al., 2012	C/C	Y	GSA	A-Phy.	Y	N	Single	Y	Y	155(3)	Y
Hillman et al., 2014	N/C	Y	GSA	P	Y	N	Single	Y	Y	221(2)	Y

*Year, Year of publication; Grp, (T/C), Baseline group imbalance (total count/conditional count); Sig., Study positivity (at least one significant test result identified by corresponding RCT); Anal., Group comparison strategy in pretest-posttest data analysis; Control, Form of control group; Random, Described random allocation procedures; Test Base, Tested baseline group equivalence on cognitive measures; Blind, Blinding procedures reported; ITT, Explicitly mentioned following intention-to-treat principle; Power, Performed a priori power analysis; N (Grp.), Total sample size (number of groups); Prereg., Pre-registered the trial. Liu-Ambrose et al. (2012) reported data dependence with Liu-Ambrose et al. (2010); E, Exercise-BS; C, Control-BS; Y, Yes; N, No; GSA, Gain score analysis; ANCOVA, Analysis of covariance; A-Cog., Active control with a cognitive focus; A-Phy., Active control with a physical focus; A-Soc., Active control with a social focus; A-Mix, Active control with more than one focus (e.g., cognitive and social); P, Passive control, Both, A control group consisting both actively and passively controlled participants; Single, Single blinding procedure (i.e., cognitive task assessors)*.

Eleven articles (33.3% of total) were randomly selected and separately coded to produce inter-coder reliability. A research assistant blinded to the study purposes completed the coding. Inter-rater reliability was calculated using Cohen's *Kappa* coefficient for each coding variable (Table 2). Following Landis and Koch's (1977) recommendations, we considered *Kappa* values between 0.61 and 0.80 as substantial and above 0.80 as very good. All the coded variables in the present study showed very good reliability. Coding discrepancies were resolved by re-visiting studies and discussion.

**Table 2:** **Kappa coefficients for coding variables**.

**Coding Variable**	**Kappa**
Cognitive task	1.00
Baseline group imbalance (Control vs. Exercise)	0.92
Group difference results (significant vs. non-significant)	1.00
Group comparison strategy (GSA vs. ANCOVA)	0.85
Form of control	1.00
Description of randomization	1.00
Baseline group equivalence test on cognitive measures	1.00
Description of blinding	0.80
Intention-to-treat principle (ITT)	1.00
*A priori* power analysis	1.00
Total participant number and number of groups	1.00
Trial pre-registration	1.00

### RCT count and statistical analysis

We categorized and counted all the RCTs regarding their group-comparison strategy and baseline group imbalance. For group-comparison strategy, we categorized a given RCT into GSA-RCT if it used *gain scores* as the criterion in comparing groups. We classified an RCT as ANCOVA-RCT if the outcome variable was the post-test score while controlling for baseline score as covariate, or if analysis of partial variance was used.

Although we coded baseline group imbalance for every cognitive task within an RCT, we later counted the number of RCT regarding their baseline group imbalance favorableness (control-BS vs. exercise-BS). This ensured an equal weight for every RCT given their varying number of cognitive measures. For example, one RCT reported 42 cognitive measures but several RCTs reported only one cognitive measure. In this case, the 42-task RCT would be over-weighted if the count were made at the task level. We applied the “dominance rule” in judging whether a given RCT favors control-BS or exercise-BS. For example, if an RCT used four cognitive measures, we coded it as favoring control-BS if three of the four measures had better performing control group at baseline. Due to within-study measurement dependence, multiple cognitive measures tended to show homogeneous results with respect to baseline group imbalance. Among 33 RCTs, we applied the dominance rule to 14 RCTs. Two RCTs showed equal number of cognitive measures between control-BS and exercise-BS, and thus were dropped from the final count on baseline group imbalance.

We also made “conditional count” among GSA-RCTs. First, all the RCTs were screened for GSA employment. Then, GSA-RCTs were categorized as either positive (i.e., having at least one significant finding) or negative (i.e., having no significant findings). The “conditional count” process was very similar to the previous count except that a RCT's baseline group imbalance was decided only on those cognitive measures fitting the positive/negative category. Specifically, if a GSA-RCT had at least one significant result (i.e., positive study), its baseline group imbalance was determined on all significant cognitive measures. If a GSA-RCT had no significant results (i.e., negative study), all its cognitive measures were included to determine its baseline group imbalance. These decisions were made for two reasons. First, some positive RCTs employed only one cognitive task (which reached statistical significance). Second, we could bias the negative RCT count regarding baseline group imbalance if we retained the non-significant measures from positive RCTs and recycled them in the negative RCT count.

During the “conditional count,” we applied the dominance rule to only one GSA-RCT because it included one cognitive measure supporting control-BS and one cognitive measure with description-wise equal baseline between the control and exercise group; and thus it was counted as control-BS. In addition, one positive GSA-RCT reported a control-BS on one cognitive measure and exercise-BS on the other cognitive measure. This RCT was subsequently classified as neutral and was dropped from the final conditional count. We used the R version 3.2.0 (R Core Team, 2015) to estimate the probability of obtaining those counts based on continuity-corrected binomial distributions. Whereas the first two hypotheses had two-sided tests, the third hypothesis had one-sided test. The alpha level was set at 0.05.

## Results

Table 3 summarizes results pertaining to the first two hypotheses. The first hypothesis assumed that the occurrence of control-BS and exercise-BS are equally likely. Among all the RCTs (*n* = 31), we observed that 16 RCTs resulted in a control-BS and 15 RCTs in an exercise-BS (two RCTs were dropped in the count because they showed no clear favorableness between control-BS and exercise-BS). The probability of detecting this result met our expectation, $\hat{k}$ = 0.52, *p* = 0.99, with a 95% CI of (0.33, 0.69). The second hypothesis assumed that the incidence of GSA and ANCOVA as a group comparison strategy are equal among RCTs. The count revealed 27 GSA-RCTs and 6 ANCOVA-RCTs. The test of such occurrence reached significance, $\hat{k}$ = 0.82, *p* < 0.001, with a 95% CI of (0.64, 0.92). Therefore, we rejected the second hypothesis and concluded that researchers predominantly used GSA over ANCOVA in analyzing pretest-posttest data.

**Table 3:** **The probability of observed RCT counts regarding baseline group imbalance and group comparison strategy**.

	**Group (*****N*** = 31**)**		**Strategy (*****N*** = 33**)**	
	**Control**	**Exercise**	**GSA**	**ANCOVA**
RCT Count	16	15	27	6
$\hat{\text{k}}$ (95% C.I.)	0.52 (0.33, 0.69)		0.82 (0.64, 0.92)	
*p*	0.99		<0.001	

*Group, Baseline group imbalance; Control, Control-BS; Exercise, Exercise-BS; Strategy, Group-comparison strategy used in pretest-posttest data analysis; GSA, Gain score analysis; ANCOVA, Analysis of covariance*.

Table 4 displays results for the third hypothesis, which tested independence between baseline group imbalance and statistical significance test result among GSA-RCTs. Among positive GSA-RCTs (*n* = 17), 14 resulted in a control-BS and three in exercise-BS. This pattern reached significant level, $\hat{k}$ = 0.82, *p* = 0.006, with a 95% CI of (0.60, 1.00). Among the negative GSA-RCTs (*n* = 9), two studies had a control-BS and seven had exercise-BS. This observation was not significant, $\hat{k}$ = 0.22, *p* = 0.09, with a 95% CI of (0.00, 0.55). Thus, baseline group imbalance was related to statistical test in that more control-BS GSA-RCTs (which had over-estimated effect sizes) than exercise-BS GSA-RCTs resulted in significant results.

**Table 4:** **The probability of observed conditional count on GSA-RCTs regarding baseline group imbalance**.

	**Positive (*****n*** = 17**)**		**Negative (*****n*** = 9**)**	
	**Control**	**Exercise**	**Control**	**Exercise**
RCT Count	14	3	2	7
$\hat{\text{k}}$ (95% C.I.)	0.82 (0.60, 1.00)		0.22 (0.00,0.55)	
*p*	0.006		0.09	

*Positive, GSA-RCTs identifying at least one significant finding; Negative, GSA-RCTs identifying no significant findings; Control, Control-BS; Exercise = Exercise-BS*.

## Discussion

The objective of the present study was to determine whether exercise-cognition RCTs published in the past 20 years (1996–2015) include false positives or false negatives due to the ignorance of Lord's paradox (i.e., performing GSA in analyzing pretest-posttest data). Overall, several findings emerged from this study. First, baseline group superiority was found to be randomly determined among all the RCTs, with an equal probability of control-BS and exercise-BS. Second, GSA was the more popular group comparison strategy (27 RCTs) compared to ANCOVA (6 RCTs). Lastly, evidence suggested that positive GSA-RCTs were likely to include false positive errors because 82% (14 out of 17 studies) of them tested on over-estimated effect sizes. However, no clear evidence supported false negative errors among negative GSA-RCTs although a descriptive consistency was revealed.

Given findings that GSA is prevalent and misleading, it is necessary to re-emphasize the adoption of ANCOVA in pretest-posttest data analysis. The employment of ANCOVA could eliminate the biased effect estimate due to baseline group imbalance and increase testing power, thus reducing inferential errors. However, choosing ANCOVA as group comparison strategy is only half the story because ANCOVA enhances causal inferences only when group equivalence is likely. The other half, baseline group equivalence, depends on multiple factors during the experimental process. Some important factors are discussed next.

### Randomization procedures

One factor influencing group equivalence is randomization procedure. According to Schulz (1996), randomization consists of two stages: generation of unpredictable assignment sequence and concealment of that sequence until group allocation occurs. The first stage is related to the reliability of the randomizing tool (e.g., computer algorithm), and is often mistakenly identified as randomization itself. Consequently, sequence-concealment often receives insufficient attention, which introduces bias that emerges from the predictability of participant allocation. Ideally, the information on participant allocation should be revealed “as late as possible.” As an example, Newell (1992) reported an anecdotal story of a surgeon who tosses a sterilized coin after a patient's abdomen was opened to decide which “treatment” he should perform. Although a little extreme, it highlights the importance of concealing participants' allocation information from experimenters. Table 1 shows that only 7 out of 33 RCTs described randomization tools and even fewer RCTs described sequence-concealment procedures. In a couple of occasions, the randomization was done with imbalanced assignment ratio (e.g., 2:1 in assigning participants to exercise and control group, respectively) and no justifications were offered. Therefore, it is encouraged to report the randomization tool and to describe procedures for concealing the randomization sequence. In cases of imbalanced group assignment ratios, justifications are required.

### Baseline check

Prior to intervention, researchers must examine group equivalence on baseline measures. To foster such an examination, the CONSORT (Consolidated Standards of Reporting Trials) statement (Schulz et al., 2010) suggests reporting baseline data of demographic and clinical characteristics for each group. Concerning the CONSORT statement and the difficulty in conducting double-blind trials in exercise-cognition area, we recommend researchers to examine baseline group equivalence using both significance tests and subjective judgments. Baseline significance tests can alert researchers to factors interfering with randomization (e.g., no double-blinding); even when no significant group differences are identified at baseline, researchers must still review descriptive group imbalance on its size and prognostic strength (Altman, 1985). If meaningful group differences are found on any of the baseline measures (regardless of test significance), researchers could take different approaches in solving the problem, depending on how many baseline measures showed group differences. For instance, researchers can block participants when only few baseline measures (i.e., one or two) showed group differences in baseline check, or can re-randomize participants when more baseline variables exhibited group differences (Rubin, 2008).

### Single-blinding and differential expectation

Blinding procedure also affects group equivalence. When participants were assigned to either exercise or control group, it was challenging (if not impossible) to blind them to their respective interventions. In the present review, 18 out of the 33 RCTs reported blinding procedures and all of them were “single-blinded” (i.e., cognitive task assessors were blinded to participants' group assignment). No RCTs reported blinding participants to their group assignments. This raises the concern that participants may show differential expectations due to open group assignment. Such a possibility is consistent with the idea of “unmatched task” for the control group in the literature dealing with the effect of exercise on cognition (Brisswalter et al., 2002). The concern of differential expectation can also be evidenced by the diversity of control conditions in Table 1. This diversity reveals little agreement among researchers in speculating an active control for exercise intervention. To help select and/or design a good control, we recommend an empirical solution. That is, researchers should measure differential expectation. Although, preliminary effort has been made to survey differential group expectations prior to intervention (e.g., Stothart et al., 2014), we echoed Boot et al. (2013) in suggesting future research to consider testing differential expectation either during or after the intervention period. The optimal active control of exercise intervention must equate expectations on all these periods.

### Intention-to-treat principle

Intention-to-Treat (ITT) is a widely accepted principle in analyzing clinical trials. ITT prevents group non-equivalence due to participant dropout (e.g., differential attrition) by including all the randomized participants in data analysis based on their intended treatment assignment (Gillings and Koch, 1991). The ideal situation for ITT would be having complete data for all the randomized participants (Hollis and Campbell, 1999). However, attrition is typically inevitable for clinical trials. In order to include participants with incomplete data into the analysis, missing values need to be handled. Some missing value imputation methods are available. For example, methods based on multiple imputation or maximum likelihood are generally recommended, but special considerations must be given to specific situations (Enders, 2010). However, no statistical methods can perfectly fix experimental flaws. When applying ITT, it is necessary to develop protocols (e.g., excluding likely exercise-intolerant participants before randomization) to ensure that participant adherence rate is roughly 80% or higher (Gillings and Koch, 1991; Montori and Guyatt, 2001). Regardless of adherence rate for a given RCT, a sensitivity test should always be performed to compare the ITT analysis results (as primary outcome) with the complete-case analysis results (Gillings and Koch, 1991). Compatible result of the sensitivity test precludes the concern of differential attrition, whereas incompatibility suggests this threat to internal validity. In short, future investigations are advised to include protocols that maximize adherence rate, to follow ITT principle, and to perform sensitivity analysis. Two other important elements of clinical trials are discussed next, although they do not affect group equivalence directly.

### Power

Despite that no clear evidence of false negative errors was observed in the present study, it was still important to make sure that each RCT has sufficient power so that false negative errors could be minimized. Among all the RCTs included, only eight of 33 RCTs reported performing an *a priori* power analysis. Depending on the inputted parameters, the sample sizes varied among these RCTs. However, the average group size among the RCTs with *a priori* power analysis was about 65 participants, whereas the average group size for those not performing an a priori power analysis was about 32 participants^3^. It seems that a substantial proportion of exercise-cognition RCTs was underpowered, and thus could lead to false negative errors. It might be argued that 23 out of 33 included RCTs had at least one significant result, and thus false negative errors should not be a concern. However, 23 out of 33 RCTs having at least one positive result is not an evidence of sufficient power. First, we showed that false positive errors are likely to be included in those 17 positive GSA-RCTs, and by extension in the 23 positive RCTs. Second, as highlighted by Rubin (1974), a poorly implemented experiment can maintain many errors and ultimately be irrelevant to testing the research question. An experiment should follow optimal procedures (including *a priori* power analysis) for its conclusions to appropriately address research questions.

### Researcher degrees of freedom and trial pre-registration

Although researchers are following the best paradigm including fixed set of practices, they still make decisions on quite some circumstances. These decision-calling circumstances are regarded as the *researcher degrees of freedom* (Simmons et al., 2011). It includes, among others, types of measure used in data collection, group-comparison strategies employed for data analysis, and type of data reported. When considering the researcher degrees of freedom with publication bias, an increased likelihood of Type I error would follow. For example, Gelman and Loken (2013) argued that data analysis strategies could be unwittingly conditioned on data patterns, which allow for false positive findings. To restrict researcher degrees of freedom by increasing clinical trial transparency, the International Committee of Medical Journal Editors (ICMJE) declared a trial's pre-registration as a condition for publishing in its 11 member journals in 2004 (De Angelis et al., 2004). ICMJE only recognizes registries meeting several criteria, including being free to public access, electronically searchable, open to all registrants, run by not-for-profit organization, as well as able to ensure validity of registration data by offering a mechanism. For example, www.clinicaltrials.gov maintained by the U.S. National Institute of Health is a qualified registry, even though many other registries have become available since 2004 (Humphreys et al., 2013) maintained by the U.S. National Institute of Health is a qualified registry, even though many other registries have become available since 2004 (Humphreys et al., 2013). It is by revealing critical trial information before participant enrollment that trial pre-registration combats researcher degrees of freedom. By pre-registering trials, researchers can still make changes afterwards as long as they offer good justifications. Although pre-registration has been the rule in clinical trial publication for almost 10 years (Laine et al., 2007), it is not true among exercise-cognition RCTs because only 8 out of 27 studies published in 2005 and later had trial pre-registration (Table 1). Therefore, we recommend future exercise-cognition RCTs to follow ICMJE's guidelines and make trial pre-registrations before enrolling participants.

### Limitations

Several limitations in the present study are worth pointing out. First, we only focused on group comparison strategies in analyzing pretest-posttest data in exercise-cognition RCTs because it generates good evidence to evaluate the claim that exercise benefits cognition, and it is a design shared by all the exercise-cognition RCTs. Second, although ANCOVA should be used in analyzing pretest-posttest data in RCTs given group equivalence, it should be noted that ANCOVA was developed under several statistical assumptions, among which the assumption of homogeneity of regression slopes should receive particular attention (Miller and Chapman, 2001). However, these assumptions should not be used as an excuse to choose GSA against ANCOVA because GSA shares the same set of assumptions and because of ANCOVA's robustness and flexibility under assumption violation (Huck and McLean, 1975). Lastly, the counting process may have introduced bias in our conclusions, especially for the conditional count. We made the counts at trial level rather than at task level, and thus applied the “dominance rule” in order to maintain equal weight among exercise-cognition RCTs. Even though a better approach may be possible, evidence supported our decision. For example, we applied the “dominance rule” only to a minority of collected RCTs and the marginal count met the exact expectation from a probability point of view. Among the 33 RCTs, only two RCTs switched the group regarding baseline superiority between the marginal count and the conditional count.

## Conclusion

Although exercise-cognition RCTs showed randomness of baseline group imbalance, RCTs adopting GSA as group comparison strategy were likely to have false positive errors and thus weakened the overall exercise-benefit-cognition claim. Future research will benefit from employing ANCOVA in analyzing pretest-posttest data while maintaining baseline group equivalence. Several suggestions have been offered to maintain baseline group equivalence in future research. It is likely that the results of current study are not limited to the effect of exercise on cognition and could potentially be extended to RCTs in other domains.

## Author contributions

Conceived and designed the study: SL, JL. Searched publications: JL. Screened publications, coded data, and analyzed results: SL. Calculated inter-rater reliability: JL. Contributed to the writing of this manuscript: SL, JL, GT.

### Conflict of interest statement

The authors declare that the research was conducted in the absence of any commercial or financial relationships that could be construed as a potential conflict of interest.

## Footnotes

^1^In this paper, the key distinction between ANCOVA and GSA is how researchers use the baseline measure. Although researchers can choose variables (e.g., age) as covariates in testing group difference on gain scores, these analyses are not what we mean by ANCOVA here.

^2^We chose *k* instead of *p* to avoid confusion later when reporting the probability of our hypothesis testing.

^3^This information was calculated based on the “*N* (Grp.)” column of Table 1.
